# Supplementary material for: Resolving the full spectrum of human genome variation using Linked-Reads
Source: Genome Res. 2019 Apr;29(4):635–45. doi: 10.1101/gr.234443.118 (PMC6442396; doi:10.1101/gr.234443.118)
Supplement: Supplemental Material [file supp_gr.234443.118_Supplemental_longranger-2.2.zip › longranger-2.2.tar.bz2!/lr2.2-public/lib/stan/cmdstan/stan/doc/api/html/check__pos__semidefinite_8hpp_source.html]

Stan: src/stan/math/error\_handling/matrix/check\_pos\_semidefinite.hpp Source File


|  |  |
| --- | --- |
|  | Stan  2.5.0  probability, sampling & optimization |


- Main Page
- Related Pages
- Namespaces
- Classes
- Files

- File List
- File Members

All Classes Namespaces Files Functions Variables Typedefs Enumerations Enumerator Friends Macros Pages

- src
- stan
- math
- error\_handling
- matrix

check\_pos\_semidefinite.hpp

Go to the documentation of this file.

1 #ifndef STAN\_\_MATH\_\_ERROR\_HANDLING\_\_MATRIX\_\_CHECK\_POS\_SEMIDEFINITE\_HPP

2 #define STAN\_\_MATH\_\_ERROR\_HANDLING\_\_MATRIX\_\_CHECK\_POS\_SEMIDEFINITE\_HPP

3

4 #include <sstream>

5

6 #include <boost/math/special\_functions/fpclassify.hpp>

7

8 #include <stan/math/error\_handling/dom\_err.hpp>

9 #include <stan/math/error\_handling/matrix/constraint\_tolerance.hpp>

10 #include <stan/math/matrix/Eigen.hpp>

11 #include <stan/math/matrix/meta/index\_type.hpp>

12

13 namespace stan {

14

15  namespace math {

16

30  // FIXME: update warnings (message has (0,0) item)

31  template <typename T\_y, typename T\_result>

32  inline bool

33  check\_pos\_semidefinite(const char\* function,

34  const Eigen::Matrix<T\_y,

35  Eigen::Dynamic,

36  Eigen::Dynamic>& y,

37  const char\* name,

38  T\_result\* result) {

39  using Eigen::Dynamic;

40  using Eigen::Matrix;

41  typedef typename index\_type<Matrix<T\_y,Dynamic,Dynamic> >::type size\_type;

42

43  if (y.rows() == 1 && !(y(0,0) >= 0.0)) {

44  std::ostringstream message;

45  message << name << " is not positive semi-definite. "

46  << name << "(0,0) is %1%.";

47  std::string msg(message.str());

48  return dom\_err(function,y(0,0),name,msg.c\_str(),"",result);

49  }

50  Eigen::LDLT< Eigen::Matrix<T\_y,Eigen::Dynamic,Eigen::Dynamic> > cholesky

51  = y.ldlt();

52  if(cholesky.info() != Eigen::Success || (cholesky.vectorD().array() < 0.0).any()) {

53  std::ostringstream message;

54  message << name << " is not positive semi-definite. "

55  << name << "(0,0) is %1%.";

56  std::string msg(message.str());

57  return dom\_err(function,y(0,0),name,msg.c\_str(),"",result);

58  }

59  for (int i = 0; i < y.size(); i++)

60  if (boost::math::isnan(y(i))) {

61  std::ostringstream message;

62  message << name << " is not positive semi-definite. "

63  << name << "(0,0) is %1%.";

64  std::string msg(message.str());

65  return dom\_err(function,y(0,0),name,msg.c\_str(),"",result);

66  }

67  return true;

68  }

69

70  }

71 }

72 #endif

boost::math::isnan

bool isnan(const stan::agrad::var &v)

Checks if the given number is NaN.

**Definition:** boost\_fpclassify.hpp:69

dom\_err.hpp

stan::math::dom\_err

bool dom\_err(const char \*function, const T &y, const char \*name, const char \*error\_msg, const T\_msg error\_msg2, T\_result \*result)

**Definition:** dom\_err.hpp:36

stan::math::check\_pos\_semidefinite

bool check\_pos\_semidefinite(const char \*function, const Eigen::Matrix< T\_y, Eigen::Dynamic, Eigen::Dynamic > &y, const char \*name, T\_result \*result)

Return true if the specified matrix is positive definite.

**Definition:** check\_pos\_semidefinite.hpp:33

constraint\_tolerance.hpp

stan::math::index\_type

Primary template class for the metaprogram to compute the index type of a container.

**Definition:** index\_type.hpp:21

index\_type.hpp

Eigen.hpp

stan::math::size\_type

index\_type< Eigen::Matrix< double, Eigen::Dynamic, Eigen::Dynamic > >::type size\_type

Type for sizes and indexes in an Eigen matrix with double e.

**Definition:** typedefs.hpp:16


---

[ Stan Home Page ]

*© 2011–2014,
Stan Development Team.*
